# IFN-Type-I Response and Systemic Immunity in Rectal Adenocarcinoma Patients Treated with Conventional or Hypofractionated Neoadjuvant Radiotherapy

**DOI:** 10.3390/biom14040448

**Published:** 2024-04-06

**Authors:** Ioannis M. Koukourakis, Erasmia Xanthopoulou, Michael I. Koukourakis, Dina Tiniakos, Vassilis Kouloulias, Anna Zygogianni

**Affiliations:** 1 Radiation Oncology Unit, 1st Department of Radiology, School of Medicine, Aretaieion Hospital, National and Kapodistrian University of Athens (NKUOA), 11528 Athens, Greece; azygogianni@med.uoa.gr; 2 Department of Radiotherapy/Oncology, Medical School, Democritus University of Thrace, 68100 Alexandroupolis, Greece; mia_x1995@hotmail.com (E.X.); mkoukour@med.duth.gr (M.I.K.); 3 Department of Pathology, Aretaieion University Hospital, School of Medicine, National and Kapodistrian University of Athens, 11528 Athens, Greece; dtiniak@gmail.com; 4 Translational and Clinical Research Institute, Faculty of Medical Sciences, Newcastle University, Newcastle upon Tyne NE2 4HH, UK; 5 Radiotherapy Unit, 2nd Department of Radiology, Attikon Hospital, School of Medicine, Rimini 1, National and Kapodistrian University of Athens, 12462 Athens, Greece

**Keywords:** radiotherapy, IFNβ, hypofractionation, rectal cancer, lymphopenia, CD8, adenocarcinoma, immunotherapy

## Abstract

The IFN-type-I pathway is involved in radiotherapy (RT)-mediated immune responses. Large RT fractions have been suggested to potently induce this pathway. Neoadjuvant hypofractionated short-course (scRT) and conventional long-course (lcRT) RT applied for the treatment of locally advanced rectal adenocarcinoma patients provides a unique model to address the immuno-stimulatory properties of RT on a systemic level. We prospectively analyzed the IFNβ plasma levels and lymphocyte counts (LCs) of rectal adenocarcinoma patients before and after treatment with scRT (n = 22) and lcRT (n = 40). Flow cytometry was conducted to assess the effects on lymphocytic subpopulations in a subset of 20 patients. A statistically significant increase in the post-RT IFNβ plasma levels was noted in patients undergoing scRT (*p* = 0.004). Improved pathological tumor regression was associated with elevated post-RT IFNβ levels (*p* = 0.003). Although all patients experienced substantial lymphopenia after treatment, the post-RT LC of patients treated with scRT were significantly higher compared to lcRT (*p* = 0.001). Patients undergoing scRT displayed significantly lower percentages of regulatory CD4+/CD25+ T-cells after therapy (*p* = 0.02). scRT enables effective stimulation of the IFN-type-I pathway on a systemic level and confers decreased lymphocytic cytotoxicity and limited regulatory T-cell activation compared to lcRT, supporting its increasing role in immuno-RT trials.

## 1. Introduction

Radiotherapy’s (RT) cytotoxic properties have been utilized since the first decades of the 20th century for cancer treatment. However, multiple studies during these early years revealed that RT has a significant interplay with the immune system that could also be exploited with the goal of improving tumor eradication [1]. Despite initial unsuccessful attempts to combine RT with the administration of cytokines, the rapid emergence of immune checkpoint molecules in oncology during the past decade has reinvigorated the interest in both pre-clinical and clinical research in the RT immuno-stimulatory properties.

Several studies have confirmed a beneficial effect in combining RT and immune checkpoint molecules [2,3]. Theelen et al. reported the data of two randomized trials on metastatic non-small cell lung carcinoma patients who received pembrolizumab with or without irradiation of 1–4 lesions [3]. The abscopal responses of non-irradiated lesions were noted in 43.4% of patients in the RT arm vs. 19.7% of patients who were treated with pembrolizumab alone. The activation of the IFN-type-I pathway has been implicated as one of the mechanisms underlying RT-mediated immune responses [4,5,6]. Specifically, IFNβ has been suggested to be the driver of the abscopal effects triggered by combining RT and immune checkpoint molecules [7]. However, limited studies are available regarding the effectiveness of RT to induce anti-tumor immunity on a systemic level. Experimental evidence highlights the importance of RT fractionation in triggering the IFN-type-I response, favoring the use of a large dose per fraction [8].

Colorectal cancer ranks third among all malignancies in terms of incidence, with rectal cancer comprising approximately 30% of the cases [9]. Ever since the early 1990s, neoadjuvant (preoperative) RT has been the standard of care for the treatment of locally advanced rectal carcinomas. In multiple randomized trials, two distinct RT schedules have been shown to confer similar benefits as far as patient survival is concerned: long-course RT (lcRT) (standard fractionated RT of 28 daily fractions of 1.8 Gy combined with chemotherapy), and short-course RT (scRT) (hypofractionated and accelerated RT of 5 daily fractions of 5 Gy, without chemotherapy) [10,11,12,13]. Moreover, total neoadjuvant therapy (TNT) has emerged as the new gold standard when treating advanced rectal carcinomas, consisting of neoadjuvant lcRT or scRT, preceded or followed by a full chemotherapy course [14]. As of recently, immunotherapy with dostarlimab, an anti-PD-1 inhibitor, is the preferred upfront treatment approach for rectal cancer patients with mismatch repair deficient tumors, with RT being prescribed in cases that do not reach clinical complete response [15]. The potential synergistic effect of concurrent scRT and immunotherapy in advanced mismatch repair proficient rectal carcinomas has been explored in the TORCH randomized phase II trial, which reported complete response rates in up to 58% of cases (preliminary results) [16]. Overall, rectal cancer provides a unique model to study the interaction of different RT schedules with the immune system.

In this study, we investigated the effects of scRT and lcRT on IFNβ plasma levels of rectal cancer patients. Moreover, we studied systemic immune responses involving changes in the regulatory and cytotoxic T-cell populations in patients undergoing neoadjuvant RT.

## 2. Materials and Methods

### 2.1. Patients

This is a collaborative prospective study between the Radiation Oncology Unit, Aretaieion Hospital, School of Medicine, National and Kapodistrian University of Athens and the Radiotherapy/Oncology Department, University Hospital of Alexandroupolis, Department of Medicine, Democritus University of Thrace. Approval has been obtained from the Ethics and Research Committees of the two hospitals (approval numbers 316/26-03-2021 and ES10/24-10-2018, respectively). The study was conducted in accordance with the Declaration of Helsinki. Inclusion criteria demanded histological biopsy confirmation of rectal adenocarcinoma, radiological documentation of locally advanced disease (extramural and/or lymph node invasion on Magnetic Resonance Imaging-MRI), good performance status (0/1) and written informed consent. Exclusion criteria concerned previous exposure to RT or chemotherapy, severe lung, kidney, heart or auto-immune disease, pregnancy, and major psychiatric disorder. Patients with any hematological disease were also excluded.

Two cohorts of consecutive patients with rectal adenocarcinoma undergoing preoperative RT were recruited for this prospective study. Patients in the first cohort (n = 40) were treated with a ‘long-course’ RT (lcRT) schedule with conventional fractionation (lcRT: 1.8 Gy/fraction x 28 fractions to a total equivalent dose delivered in 2 Gy fractions-EQD2-of 48.96 Gy delivered within 38 days). Patients in the second cohort (n = 22) were treated with a hypofractionated ‘short-course’ RT (scRT) schedule (scRT: 5 Gy/fraction × 5 fractions to a total EQD2 of 36 Gy within 5 days, corresponding to an estimated time corrected EQD2-T of 44 Gy for a λ-value of 0.4 Gy/day). A simultaneously integrated boost (SIB) of 0.2 Gy was applied to the radiologically detectable rectal mass, increasing the EQD2-T to 48 Gy. We calculated the EQD2 using the following formula: EQD2 = Total dose × [α/β + dose per fraction]/[α/β + 2]. For rectal adenocarcinoma damage, we considered an α/β of 5 Gy [17]. The EQD2 with time correction was calculated with the following formula: EQD2-T = EQD2 + λ(Δt), where ‘Δt’ is the gain in terms of number of days demanded to deliver the same EQD2 with conventional fractionation, and ‘λ’ is the estimated daily radiation dose consumed to compensate for tumor repopulation [18]. A comparison of the demographics of the two patient cohorts showed no statistically significant differences (Supplementary Table S1).

Patients in the lcRT cohort received daily oral capecitabine (825 mg/m^2^ twice a day, 5 days a week) for 5 weeks starting on day 1 of RT. Patients in the scRT cohort received FOLFOX chemotherapy in the context of total neoadjuvant therapy.

Pathological tumor regression grade (TRG) in the resection specimens was centrally assessed using the American Joint Committee on Cancer–College of American Pathologists (AJCC-CAP) system [19].

### 2.2. Plasma and Peripheral Blood Mononuclear Cells Collection

Ten ml of venous blood were collected in vials containing ethylenediaminetetraacetic acid (EDTA). This was performed at the beginning of therapy (day 1 of RT) and one week after the last fraction of RT.

Plasma and peripheral blood mononuclear cells (PBMCs) were isolated after density gradient centrifugation using a 1.077 g/mL synthetic epichlorohydrin sucrose polymer Lymphosep [Lymphocyte Separation Media-500 mL; density 1077 g/mL; Cat no: LM-T1702/500; Biosera, Cholet, France], at a 1:1 ratio of the total blood volume. Centrifugation of blood samples was performed at 1500 rpm (30 min, 25 °C). The plasma layer was transferred in sterile Eppendorf tubes and was gradually frozen at −20 °C and subsequently at −80 °C. The PBMC layer was also collected for further studies and stored at −20 °C.

### 2.3. ELISA

An ELISA assay was performed for the detection of plasma INFβ, following the instructions of the assay manual. Initially, standard curves were created using different dilutions of IFNβ (Supplementary Figure S1a). Tests were performed in duplicates.

The same ELISA technique was applied for the supernatant of two colon cancer cell lines (HT-29 and Caco-2) cultured in 6-well plates and irradiated with one fraction of 0, 4, and 10 Gy (Supplementary Figure S1b). The supernatant was collected 72 h after irradiation and ELISA was performed. The irradiation experiments were conducted in triplicates and the mean values with standard deviations from the three experiments were calculated.

Briefly, the IFNβ levels were detected with the Human Interferon beta SimpleStep ELISA^®^ Kit (ab278127, Cambridge, UK) with a standard curve ranging between 0 and 1200 pg/mL. 50 μL per sample were used in duplicates for standard and test samples.

The optical density (OD) absorbance at 450 nm was read using an OMEGA Microplate reader for all ELISA assays. The relative OD 450 nm was calculated using the following equation: Relative OD 450 = (the OD 450 of each well) − (the OD 450 of blank well).

### 2.4. Flow Cytometry

Flow cytometry for lymphocyte subset analysis was performed in two cohorts (lcRT vs. scRT) of 10 patients each, before and after RT. The lymphocyte counts (LCs) were recorded before flow cytometry.

PBMCs were isolated via differential density centrifugation with Lymphosep [Lymphocyte Separation Media-500 mL; density 1077 g/mL; Cat no: LM-T1702/500; Biosera, Cholet, France]. The experimental procedure for lymphocyte staining was based on the use of the FoxP3 Staining Kit [Cat no: 560133; Becton, Dickinson (BD), Franklin Lakesd, NJ, USA]. PBMCs were primarily stained with the CD4-FITC antibody, the CD25-APC antibody [FoxP3 Staining Kit; Cat no: 560133; Becton, Dickinson (BD), USA], the CD8- PerCP-Cy5.5 antibody [CD8 Mouse Human PerCP-Cy5.5; Cat no: 560662; Becton, Dickinson (BD), USA], and the CD3-BV421 antibody [Brilliant Violet 421™ anti-human CD3 Antibody; Clone: OKT3; Cat no: 317344; BioLegend, San Diego, CA, USA], for 30 min at room temperature. Afterwards, cells were centrifuged at 850× *g* for 5 min, washed, fixed and permeabilized with 1× Human FoxP3 Buffer A (10×) for 15 min, and 1× working solution Human FoxP3 Buffer C (50×) [FoxP3 Staining Kit; Cat no: 560133; Becton, Dickinson (BD), USA] for 30 min, respectively, according to the manufacturer. Subsequently, addition of the FoxP3 [FoxP3 Staining Kit; Cat no: 560133; Becton, Dickinson (BD), USA] antibody was performed and the cells were stained for 30 min, and then were centrifuged at 850× *g* for 5 min, washed and redissolved in PBS 1× solution [Biosera, FR, Lot no: 017BS454].

The BD FACSAria™ III system was utilized to analyze all samples, and subsequently, the data were analyzed using the FlowJo V10 software. Compensation for each fluorochrome was performed prior to the analysis and the voltages of each laser were adjusted based on unstained, single-stained, and multi-stained samples. The gating strategy applied was based on the unstained samples and according to the manufacturer. Recording of the events of each sample was set at either 30.000 or 50.000 events based on the quality of the sample.

### 2.5. Cell Line Experiments

To examine whether rectal cancer cell irradiation results in the release of IFNβ, we performed in vitro experiments with two colorectal adenocarcinoma cell lines, HT-29 and Caco-2, purchased from the American Type Culture Collection (ATCC^®^ HTB-38™ and ATCC^®^ HTB-37™, respectively, Manassas, VA, USA). Cell line characteristics and authentication are available at https://www.atcc.org/products/htb-38 and at https://www.atcc.org/products/htb-37 (Accessed on 2 February 2024). Briefly, the Caco-2 cell line is a human colorectal cancer cell line that was first obtained from a 72-year-old Caucasian man. The HT-29 human cell line was originally derived from a 44-year-old Caucasian woman with colorectal adenocarcinoma. All cell lines were tested for mycoplasma before the experiments were conducted, and further authentication has also been obtained by Eurofins Genomics (Ebersberg, Gemany).

Cell lines were cultured under aseptic conditions, using Dulbecco’s Modified Eagle Medium (DMEM Low Glucose) [Material: LM-D1102/500; Batch no: MS008W; ID no: MS008W100R; Biosera, Cholet, France], enriched with 10% (*v*/*v*) fetal bovine serum [Cat no: FB-1001/500; Cholet, France] and supplemented with 1% (*v*/*v*) penicillin–streptomycin buffer [Cat no: LM-A4118/100; Cholet, France].

Cell lines were cultured in standard conditions in 6-well plates in a 5% CO_2_ incubator at 37 °C, a day prior to the irradiation treatment. Plates were irradiated with a 6MV LINAC (PRECISE, ELEKTA) to receive 0, 4, and 10 Gy in one fraction. The supernatant of the cell cultures was collected 72 h after irradiation, and the IFNβ levels were measured with ELISA, as reported above.

### 2.6. Statistical Analysis

We used the GraphPad Prism 8.0.2 statistical package for statistical analysis and graph presentation. Box and whisker plots show the range, median values, and 25th and 75th percentiles. The Wilcoxon matched pairs signed rank test was used to test for significant differences between paired groups of continuous variables. The Mann–Whitney test was utilized to compare unpaired groups of continuous variables. A *p*-value < 0.05 was considered significant.

## 3. Results

### 3.1. Cell Line Experiments

**Figure 1:**
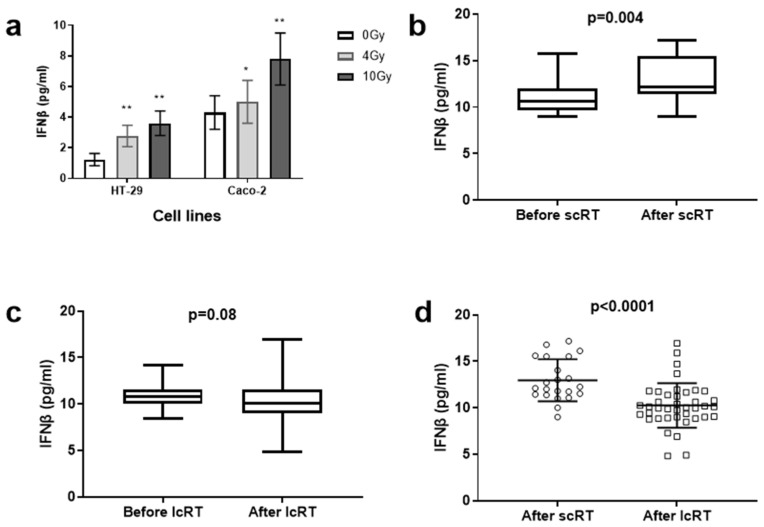
Changes in the mean (±standard deviation) concentration of IFNβ in the supernatant of colorectal cancer cell lines following irradiation with 4 and 10 Gy (* *p*-value < 0.05, ** *p*-value < 0.01) (**a**). Graph presentation of plasma IFNβ levels before and after radiotherapy (RT) in patients treated with short-course (scRT) (**b**) and long-course (lcRT) (**c**) RT. Comparison of the IFNβ levels post-RT in the two groups (**d**). Box and whisker plots show the median value, range, and the 25th and 75th percentiles. Scatter plot shows individual values and mean ± standard deviation.

Irradiation of cancer cell lines resulted in a clear increase in IFNβ in the supernatant (*p* < 0.01), which was more intense after the administration of the higher 10 Gy radiation dose (*p* < 0.01) (Figure 1a). For the HT-29 cell line, the mean ± standard deviation values for the IFNβ concentration (pg/mL) were 1.22 ± 0.4, 2.76 ± 0.7 and 3.6 ± 0.8 in control and cells treated with 4 and 10 Gy, respectively. A similar increase was noted in the Caco-2 cell line, where the IFNβ concentration was 4.3 ± 1.1, 5 ± 1.4 and 7.8 ± 1.7, respectively.

### 3.2. Plasma IFNβ Levels in the Two Cohorts

Analysis of the plasma levels in patients treated with scRT showed a significant increase in IFNβ plasma levels (pg/mL) at the end of RT (median/mean ± standard deviation 10.6/11.1 ± 1.7 vs. 12.2/10.9 ± 2.2, *p* = 0.004) (Figure 1b). In contrast, patients treated with lcRT displayed no significant changes in IFNβ levels (10.7/10.9 ± 1.2 vs. 10.1/10.2 ± 2.4, *p* = 0.08) (Figure 1c). Moreover, IFNβ plasma levels after RT were significantly higher in patients who underwent scRT vs. lcRT (*p* < 0.0001) (Figure 1d). Eight out of 22 (36%) patients in the scRT group experienced a more than 30% (30–62%) increase in IFNβ levels vs. 4 out of 40 (10%) patients in the lcRT cohort (30–37% increase).

### 3.3. IFNβ Levels and Tumor Regression Grade

**Figure 2:**
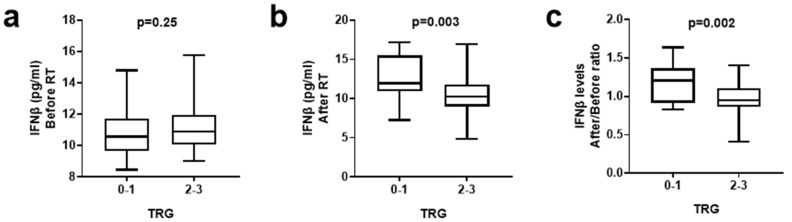
IFNβ levels before (**a**) and after (**b**) radiotherapy (RT), and IFNβ levels after/before RT ratio (**c**), stratified for tumor regression grade (TRG). Box and whisker plots show the median value, range, and the 25th and 75th percentiles.

We further compared the IFNβ plasma levels (pg/mL) in two groups of patients according to TRG: TRG 0, 1 (18 patients) vs. TRG 2, 3 (44 patients). Looking into the IFNβ levels before treatment, we found no statistical difference between the TRG groups (median/mean ± standard deviation 10.5/10.7 ± 1.5 vs. 10.9/11.1 ± 1.4, *p* = 0.25) (Figure 2a). In contrast, the IFNβ levels post-RT were significantly higher in the TRG 0, 1 patient subgroup (11.9/12.7 ± 2.7 vs. 10.2/10.6 ± 2.4, *p* = 0.003) (Figure 2b). In addition, the ratio of IFNβ levels after/before RT was significantly elevated in patients with TRG 0, 1 (1.2/1.2 ± 0.2 vs. 0.9/0.9 ± 0.2, *p* = 0.002) (Figure 2c).

### 3.4. Lymphocyte Count Analysis

**Figure 3:**
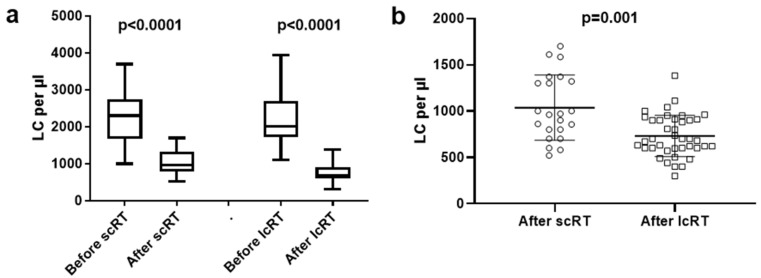
Graph presentation of lymphocyte counts (LCs) before and after short-course (scRT) and long-course (lcRT) radiotherapy (RT) (**a**), and comparison of LCs after RT between the two schedules (**b**). Box and whisker plots show the median value, range, and the 25th and 75th percentiles. Scatter plot shows individual values and mean ± standard deviation.

Analysis of the LCs displayed a strong lymphotoxic effect of RT (median/mean ± standard deviation 2303/2230 ± 717 vs. 965/1036 ± 352 before and after scRT, respectively, *p* < 0.0001; 2008/2239 ± 680 and 690/730 ± 223 before and after lcRT, respectively, *p* < 0.0001) (Figure 3a). Comparison of the post-RT LCs between the two groups revealed significantly more profound lymphopenia in the lcRT cohort (*p* = 0.001); Figure 3b.

### 3.5. CD4/CD8 Lymphocyte Subset Analysis

LC assessment preceded flow cytometry performed in 20 patients. Similarly to the overall analysis, LCs significantly decreased regardless of the schedule of RT. In the lcRT group, LCs dropped from a median of 2250/μL to 690/μL (*p* = 0.002), while in the scRT group, LCs declined from 2500/μL to 955/μL (*p* = 0.002). Nevertheless, the reduction in LC was significantly more profound in the lcRT than the scRT schedule (*p* = 0.03).

**Figure 4:**
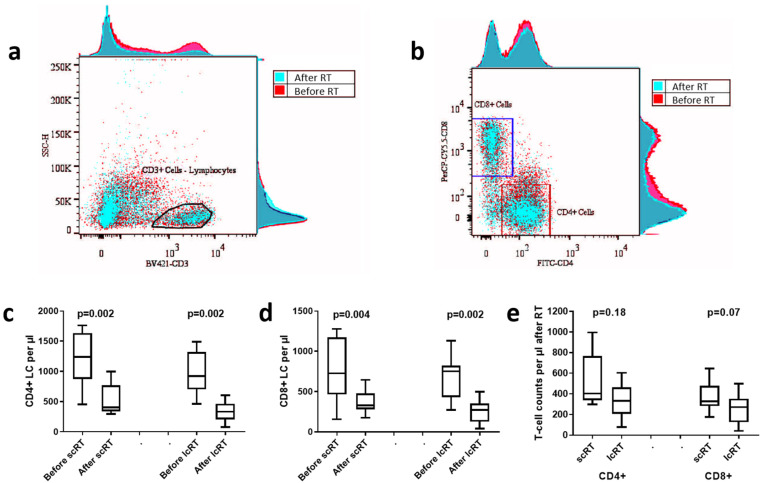
Typical flow cytometry images showing the CD3+ cell (pan T-cell marker) population (**a**) and the CD4+, and CD8+ T-cell subpopulations in CD3+ T-cells (**b**), before (red) and after (blue) radiotherapy (RT). Graph presentation of CD4+ lymphocyte counts (LCs) per μL (**c**), CD8+ LC per μL (**d**) before and after short-course (scRT) and long-course (lcRT) radiotherapy (RT). (**e**) shows the comparison of the two T-cell subpopulation counts after scRT and lcRT. Box and whisker plots show the median value, range, and the 25th and 75th percentiles.

Flow cytometry analysis of lymphocyte subsets was performed to detect changes in the CD4+ (memory or regulatory) and CD8+ (cytotoxic) T-cells after RT. The percentage was calculated in the total T-cell population, as assessed with CD3 pan-T-cell staining. No significant changes after RT were noted, for both CD4+ T-cells (median/mean ± standard deviation 43.2/47 ± 8.7 vs. 45.5/45.1 ± 11.1 for scRT, *p* = 0.75; 42/40.8 ± 8.2 vs. 43.6/41.4 ± 9.5 for lcRT, *p* = 0.92) and CD8+ T-cells (31.9/30 ± 9.2 vs. 33.8/32.1 ± 6.1 for scRT, *p* = 0.11; 28.4/28.3 ± 9.2 vs. 27.8/30.3 ± 12.3 for lcRT, *p* = 0.25), regardless of the RT schedule. The CD4/CD8 T-cell ratio also remained at the same levels (1.5/1.8 ± 0.9 vs. 1.2/1.4 ± 0.5 for scRT, *p* = 0.08; 1.5/1.5 ± 0.3 vs. 1.4/1.5 ± 0.5 for lcRT, *p* = 0.69). Figure 4a,b present typical flow cytometry images showing the CD3+ cell population (pan T-cell marker) and the CD4+, along with the CD8+ T-cell subpopulations in CD3+ T-cells before and after RT.

We further assessed the absolute CD4+ and CD8+ T-cell counts by extrapolating their percentage values to the total LC assessed in the corresponding blood count examination (Figure 4c–e). The absolute values of CD4+ T-cells significantly decreased after scRT (median/mean ± standard deviation 1238/1205 ± 442 vs. 403/507 ± 243, *p*-value = 0.002) and lcRT (920/983 ± 340 vs. 332/336 ± 171, *p* = 0.002; Figure 4c). Similarly, CD8+ T-cells significantly decreased after scRT (727/765 ± 367 vs. 328/367 ± 145, *p* = 0.004) and lcRT (750/680 ± 265 vs. 270/246 ± 139, *p* = 0.002) (Figure 4d). Comparing the CD4+ and CD8+ LCs after RT (Figure 4e), it was found that patients treated in the scRT had marginally higher absolute numbers of CD8+ T-cells (*p* = 0.07). CD4+ T-cells did not reach statistical significance (*p* = 0.18).

### 3.6. CD25+ and FOXP3+ Regulatory T-Cell Subset Analysis

**Figure 5:**
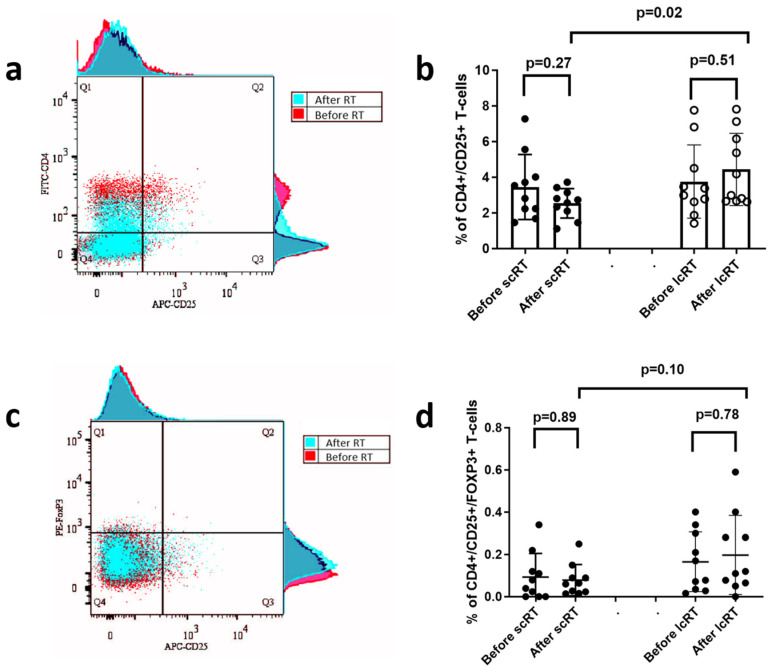
Typical flow cytometry image showing the CD4+/CD25+ T-cell population in CD3+ T-cells, before (red) and after (blue) radiotherapy (RT) (**a**). Graph presentation of the percentage of CD4+/CD25+ T-cells in CD3+ T-cells before and after short-course (scRT) and long-course (lcRT) RT (**b**). Typical flow cytometry image showing the CD25+ and FOXP3+ T-cell subpopulations in CD4+ T-cells, before (red) and after (blue) radiotherapy (RT) (**c**). Graph presentation of the percentage of CD4+/CD25+/FOXP3+ T-cells in CD3+ T-cells before and after short-course (scRT) and long-course (lcRT) RT (**d**). Box and whisker plots show the median value, range, and the 25th and 75th percentiles.

The percentage of CD4+ T-cells expressing CD25+ (a subset of regulatory T-cells) in the total CD3+ T-cell population was examined before and after RT. Figure 5a demonstrates a typical flow cytometry image showing the CD4+/CD25+ T-cell population in CD3+ T-cells, before and after RT. Τhere was no significant change in the percentage of CD4+/CD25+ T-cells in the two time points. However, this percentage was significantly lower after RT in patients treated with scRT compared to lcRT (median/mean ± standard deviation 2.6/2.5 ± 0.8 vs. 3.6/4.4 ± 2, *p*-value = 0.02) (Figure 5b).

We also assessed the effects of the two RT schedules on the regulatory CD4+/CD25+/FOXP3+ T-cell population. Figure 5c demonstrates a typical flow cytometry image showing the CD4+/CD25+/FOXP3+ T-cell population in CD3+ T-cells, before and after RT. The percentage of this subpopulation was low in both groups of patients and remained low after the end of RT. This percentage after RT was marginally lower in patients treated with scRT compared to lcRT (median/mean ± standard deviation 0.06/0.08 ± 0.07 vs. 0.11/0.19 ± 0.18, *p*-value = 0.10) (Figure 5d).

## 4. Discussion

Type-I IFNs (α, β, ε, κ and ω) are the largest IFN group in humans [20]. They have direct effects on cancer cells, inducing apoptosis and cell-cycle arrest, but also on stromal dendritic cell activation and subsequent T-cell priming following their migration to the lymph nodes [21]. Radiation-induced activation of the IFN-type-I pathway in cancer cells has been recently confirmed in several studies, suggesting an important interplay between RT and the immune system. Deng et al. showed that cancer cell irradiation promotes cytosolic DNA-sensing by cyclic GMP-AMP synthase (cGAS) followed by a STING-dependent IFN-type-I response [4]. Nevertheless, a STING-independent mechanism of IFN-type-I response has also been postulated [22]. In addition, Vanpouille-Box et al. showed in vitro that 3 fractions of 8 Gy led to increased IFNβ secretion in breast and colorectal cancer [5]. A more recent study by Jin et al. reported enhanced IFNβ expression in head and neck cancer cells and melanoma tumors implanted in mice, post-treatment with RT (1 fraction of 8 Gy) and an ataxia-telangiectasia mutated inhibitor [23]. We recently demonstrated that neoadjuvant RT of rectal adenocarcinoma patients leads to an increased expression in IFNβ and STING by cancer cells, in parallel with a restoration of the expression of HLA-class-I molecules [6]. Thus, it is implied that primary tumor irradiation triggers an in situ anti-tumor immune response and enhanced recognition of cancer cells by T-cells, which could lead to improved tumor eradication and disease control.

Nevertheless, Spitzer et al. reported that an effective immune response in the tumor microenvironment demands persistent systemic immune cell activation and proliferation [24]. The tumor microenvironment should be continually supplied with peripheral activated T-cells, as the adverse intratumoral conditions quickly set T-cells into an exhausted state. Whether the IFN-type-I response triggered by local tumor irradiation can also activate and sustain systemic anti-tumor activity is unclear. The herein-confirmed release of IFNβ by irradiated colorectal cancer cell lines in the culture medium strongly suggests that IFN-type-I cytokines may well enter the blood stream and contribute to immune system activation. We therefore investigated whether IFNβ levels increase in the plasma of rectal adenocarcinoma patients treated with neoadjuvant RT. As the IFNβ release by irradiated cancer cells appears RT-dose dependent, we further investigated whether large RT fractions, similar to those used in the hypofractionated scRT schemes, may better exploit this important immunostimulatory pathway compared to smaller fractions applied in lcRT schedules.

Indeed, in the current study, the plasma IFNβ levels of patients who underwent neoadjuvant scRT significantly increased after therapy, a finding that was not confirmed in the lcRT patient subgroup, where IFNβ levels remained unaltered. Moreover, the IFNβ levels post-RT of patients who received the hypofractionated schedule were significantly more elevated when compared to lcRT patients. Analysis according to TRG showed a statistically significant association between higher IFNβ levels and more extensive pathological tumor regression. These findings support the stimulating role of large RT fractions in the IFN-type-I response, and the eventual significance of an enhanced anti-tumor immune response in disease regression. A small number of studies have evaluated IFN levels in the peripheral blood before and after RT. Formenti et al. investigated the potential mechanism through which combination of RT (6 Gy × 5 or 9 Gy × 3 delivered to 1 metastatic site) and ipilimumab, an anti-CTLA-4 monoclonal antibody, can trigger abscopal responses in metastatic non-small cell lung carcinoma patients [7]. Interestingly, serum IFNβ levels were significantly increased only in patients who responded to combined treatment, and in patients with stable disease, to a lesser degree. No substantial changes were noted in patients who progressed during treatment. Authors suggested that induction of IFNβ could mediate an effective anti-tumor response.

We have confirmed an important lymphotoxic effect of RT. Nevertheless, LCs in patients treated with scRT were overall higher than the lcRT subgroup after treatment completion. It is well known that RT-induced lymphopenia is a crucial adverse event that patients face during treatment that potentially hinders an effective immune response against cancer cells [25,26]. Overall, our findings are suggestive of a stronger anti-tumor immune response through enhanced IFNβ release with hypofractionated RT schedules that are simultaneously less cytotoxic to circulating lymphocytes.

To assess whether RT through IFN-type-I response induction or other pathways induced changes in the peripheral blood lymphocyte subpopulations, we performed a flow cytometry analysis of T-cells in 2 cohorts of 10 patients treated with scRT and lcRT. CD8+ T-cells, through their interaction with antigen presenting dendritic cells, exert their cytotoxic properties against cancer, while CD4+ cells can either contribute to CD8+ T-cell activation (T helper cells-Th) or, following differentiation from regulatory T-cells (Tregs), suppress CD8+ T-cell activity. CD4+ regulatory T-cells are usually characterized by CD25 ± FOXP3 expression [27]. An et al. showed that CD4+/CD25+/FOXP3+ regulatory T-cells were significantly increased in patients with extensive stage small cell lung carcinoma when compared to controls, while an enhanced proliferation potential of CD8+ T-cells was indicative of better progression-free survival [28]. In addition, lower overall T-cell and cytotoxic/helper T-cell counts were noted in colorectal cancer patients vs. healthy controls [29].

In our study, no significant changes were noted in CD4+ and CD8+ T-cells percentages after RT; their absolute counts, however, drastically declined, as expected due to the overall decrease in LCs. Patients within the scRT group displayed marginally higher CD4+ and CD8+ T-cell counts after treatment when compared to lcRT patients. While no differences in the percentages of CD4+/CD25+ regulatory T-cells before and after RT in either schedule were evident, this percentage after RT was significantly higher in patients undergoing lcRT vs. scRT. In agreement with the above, the percentage of CD4+/CD25+ cells expressing FOXP3 after RT was marginally lower in patients undergoing scRT. These findings converge to an additional benefit from scRT that seems to prevent overactivation of immunosuppressive T-cell pathways in a patient subset.

A survey of the literature revealed a small number of studies focusing on T-cell subpopulations and RT. Wang et al. reported that a median dose of 60 Gy delivered to the mediastinum of 72 non-small cell lung carcinoma patients was associated with a significant increase in peripheral CD8+ T-cells [30]. Furthermore, stereotactic ablative RT of 3 fractions of 15–20 Gy or 4 fractions of 13.5 Gy delivered to 46 patients with liver and lung metastases was shown to elevate the number of circulating CD8+ T-cells and suppress CD4+/CD25+ regulatory T-cells [31]. Significant alterations in the T-cell receptor repertoire during combined RT with ipilimumab and their association with treatment response are also indicative of the complex interplay between RT and the immune system [7].

Although a larger number of patients is required to extract solid conclusions, especially in the flow cytometry analysis, the prospective nature of the study is an advantage that increases the significance of the results. The impact of IFNβ and lymphocyte subpopulations levels on locoregional control and patient survival should be sought in multi-center prospective trials with long follow-up.

## 5. Conclusions

Although there is no clear evidence regarding the superiority of either RT schedule for the treatment of locally advanced rectal carcinomas, the presented data indicate that large RT fractions delivered through scRT can more potently induce an anti-tumor immune response through the IFN-type-I pathway compared to standard fractionation (lcRT). This has been confirmed by the higher IFNβ levels of patients post-scRT. Moreover, scRT appears to confer lower lymphotoxicity, and limit the immunosuppressive effects of regulatory T-cells. Thus, in the context of immune interactions, scRT is suggested to be a better candidate when considering the incorporation of immunotherapy agents in the already established treatment practice. Nevertheless, different pathways can also be involved in this RT-mediated immune system stimulation, and further studies are required towards this direction in order to better exploit the beneficial effects of RT and immunotherapy combinations for cancer treatment.

## Data Availability

Research data are stored in an institutional repository and will be shared upon reasonable request to the corresponding author.

## Supplementary Materials

The following supporting information can be downloaded at: https://www.mdpi.com/article/10.3390/biom14040448/s1, Table S1—Patient and disease characteristics according to the radiotherapy regimen (short-course radiotherapy—scRT and long-course radiotherapy—lcRT). Figure S1—Standard ELISA curves of IFNβ concentration vs. absorbance. (a) ELISA curve for the IFNβ plasma levels and (b) ELISA curve for the concentration of IFNβ in the supernatant of cancer cells.

## References

[1] Koukourakis I.M., Koukourakis M.I. (2023). Combining the past and present to advance immuno-radiotherapy of cancer. Int. Rev. Immunol..

[2] Spigel D.R., Faivre-Finn C., Gray J.E., Vicente D., Planchard D., Paz-Ares L., Vansteenkiste J.F., Garassino M.C., Hui R., Quantin X. (2022). Five-Year Survival Outcomes from the PACIFIC Trial: Durvalumab after Chemoradiotherapy in Stage III Non-Small-Cell Lung Cancer. J. Clin. Oncol..

[3] Theelen W., Chen D., Verma V., Hobbs B.P., Peulen H.M.U., Aerts J., Bahce I., Niemeijer A.L.N., Chang J.Y., de Groot P.M. (2021). Pembrolizumab with or without radiotherapy for metastatic non-small-cell lung cancer: A pooled analysis of two randomised trials. Lancet Respir. Med..

[4] Deng L., Liang H., Xu M., Yang X., Burnette B., Arina A., Li X.D., Mauceri H., Beckett M., Darga T. (2014). STING-Dependent Cytosolic DNA Sensing Promotes Radiation-Induced Type I Interferon-Dependent Antitumor Immunity in Immunogenic Tumors. Immunity.

[5] Vanpouille-Box C., Alard A., Aryankalayil M.J., Sarfraz Y., Diamond J.M., Schneider R.J., Inghirami G., Coleman C.N., Formenti S.C., Demaria S. (2017). DNA exonuclease Trex1 regulates radiotherapy-induced tumour immunogenicity. Nat. Commun..

[6] Koukourakis I.M., Xanthopoulou E., Sgouras T.I., Kouroupi M., Giatromanolaki A., Kouloulias V., Tiniakos D., Zygogianni A. (2023). Preoperative chemoradiotherapy induces multiple pathways related to anti-tumour immunity in rectal cancer. Br. J. Cancer.

[7] Formenti S.C., Rudqvist N.P., Golden E., Cooper B., Wennerberg E., Lhuillier C., Vanpouille-Box C., Friedman K., Ferrari de Andrade L., Wucherpfennig K.W. (2018). Radiotherapy induces responses of lung cancer to CTLA-4 blockade. Nat. Med..

[8] Demaria S., Guha C., Schoenfeld J., Morris Z., Monjazeb A., Sikora A., Crittenden M., Shiao S., Khleif S., Gupta S. (2021). Radiation dose and fraction in immunotherapy: One-size regimen does not fit all settings, so how does one choose?. J. Immunother. Cancer.

[9] Siegel R.L., Giaquinto A.N., Jemal A. (2024). Cancer statistics, 2024. CA Cancer J. Clin..

[10] Bujko K., Wyrwicz L., Rutkowski A., Malinowska M., Pietrzak L., Krynski J., Michalski W., Oledzki J., Kusnierz J., Zajac L. (2016). Long-course oxaliplatin-based preoperative chemoradiation versus 5 × 5 Gy and consolidation chemotherapy for cT4 or fixed cT3 rectal cancer: Results of a randomized phase III study. Ann. Oncol..

[11] Ngan S.Y., Burmeister B., Fisher R.J., Solomon M., Goldstein D., Joseph D., Ackland S.P., Schache D., McClure B., McLachlan S.A. (2012). Randomized trial of short-course radiotherapy versus long-course chemoradiation comparing rates of local recurrence in patients with T3 rectal cancer: Trans-Tasman Radiation Oncology Group trial 01.04. J. Clin. Oncol..

[12] Erlandsson J., Holm T., Pettersson D., Berglund A., Cedermark B., Radu C., Johansson H., Machado M., Hjern F., Hallbook O. (2017). Optimal fractionation of preoperative radiotherapy and timing to surgery for rectal cancer (Stockholm III): A multicentre, randomised, non-blinded, phase 3, non-inferiority trial. Lancet Oncol..

[13] Koukourakis I.M., Kouloulias V., Tiniakos D., Georgakopoulos I., Zygogianni A. (2023). Current status of locally advanced rectal cancer therapy and future prospects. Crit. Rev. Oncol. Hematol..

[14] Bahadoer R.R., Dijkstra E.A., van Etten B., Marijnen C.A.M., Putter H., Kranenbarg E.M., Roodvoets A.G.H., Nagtegaal I.D., Beets-Tan R.G.H., Blomqvist L.K. (2021). Short-course radiotherapy followed by chemotherapy before total mesorectal excision (TME) versus preoperative chemoradiotherapy, TME, and optional adjuvant chemotherapy in locally advanced rectal cancer (RAPIDO): A randomised, open-label, phase 3 trial. Lancet Oncol..

[15] Cercek A., Lumish M., Sinopoli J., Weiss J., Shia J., Lamendola-Essel M., El Dika I.H., Segal N., Shcherba M., Sugarman R. (2022). PD-1 Blockade in Mismatch Repair-Deficient, Locally Advanced Rectal Cancer. N. Engl. J. Med..

[16] Wang Y.Q., Shen L.J., Wan J.F., Zhang H., Wang Y., Wu X., Wang J.W., Wang R.J., Sun Y.Q., Tong T. (2023). [Short-course radiotherapy combined with CAPOX and PD-1 inhibitor for the total neoadjuvant therapy of locally advanced rectal cancer: The preliminary single-center findings of a prospective, multicentre, randomized phase II trial (TORCH)]. Zhonghua Wei Chang. Wai Ke Za Zhi.

[17] Suwinski R., Wzietek I., Tarnawski R., Namysl-Kaletka A., Kryj M., Chmielarz A., Wydmanski J. (2007). Moderately low alpha/beta ratio for rectal cancer may best explain the outcome of three fractionation schedules of preoperative radiotherapy. Int. J. Radiat. Oncol. Biol. Phys..

[18] Koukourakis M.I., Damilakis J. (1994). LQ-based model for biological radiotherapy planning. Med. Dosim..

[19] Mace A.G., Pai R.K., Stocchi L., Kalady M.F. (2015). American Joint Committee on Cancer and College of American Pathologists regression grade: A new prognostic factor in rectal cancer. Dis. Colon Rectum.

[20] Fenton S.E., Saleiro D., Platanias L.C. (2021). Type I and II Interferons in the Anti-Tumor Immune Response. Cancers.

[21] Koukourakis M.I., Giatromanolaki A. (2022). Tumor draining lymph nodes, immune response, and radiotherapy: Towards a revisal of therapeutic principles. Biochim. Biophys. Acta Rev. Cancer.

[22] Goedegebuure R.S.A., Kleibeuker E.A., Buffa F.M., Castricum K.C.M., Haider S., Schulkens I.A., Ten Kroode L., van den Berg J., Jacobs M., van Berkel A.M. (2021). Interferon- and STING-independent induction of type I interferon stimulated genes during fractionated irradiation. J. Exp. Clin. Cancer Res..

[23] Jin W.J., Zangl L.M., Hyun M., Massoud E., Schroeder K., Alexandridis R.A., Morris Z.S. (2023). ATM inhibition augments type I interferon response and antitumor T-cell immunity when combined with radiation therapy in murine tumor models. J. Immunother. Cancer.

[24] Spitzer M.H., Carmi Y., Reticker-Flynn N.E., Kwek S.S., Madhireddy D., Martins M.M., Gherardini P.F., Prestwood T.R., Chabon J., Bendall S.C. (2017). Systemic Immunity Is Required for Effective Cancer Immunotherapy. Cell.

[25] Sun G.Y., Wang S.L., Song Y.W., Jin J., Wang W.H., Liu Y.P., Ren H., Fang H., Tang Y., Zhao X.R. (2020). Radiation-Induced Lymphopenia Predicts Poorer Prognosis in Patients with Breast Cancer: A Post Hoc Analysis of a Randomized Controlled Trial of Postmastectomy Hypofractionated Radiation Therapy. Int. J. Radiat. Oncol. Biol. Phys..

[26] Ellsworth S.G. (2018). Field size effects on the risk and severity of treatment-induced lymphopenia in patients undergoing radiation therapy for solid tumors. Adv. Radiat. Oncol..

[27] Gao R., Shi G.P., Wang J. (2022). Functional Diversities of Regulatory T Cells in the Context of Cancer Immunotherapy. Front. Immunol..

[28] An N., Wang H., Jia W., Jing W., Liu C., Zhu H., Yu J. (2019). The prognostic role of circulating CD8(+) T cell proliferation in patients with untreated extensive stage small cell lung cancer. J. Transl. Med..

[29] Zhang L., Chen X., Zu S., Lu Y. (2022). Characteristics of circulating adaptive immune cells in patients with colorectal cancer. Sci. Rep..

[30] Wang H., Li Y., Hu P., Zhang J. (2024). The Correlation Between Low-Dose Radiotherapy Area of the Mediastinum and CD8+T Cells and the Efficacy of Radiotherapy for Non-Small Cell Lung Cancer. Cancer Manag. Res..

[31] Novikov S.N., Baldueva I.A., Zozulya A.Y., Emelyanova N.V., Girdyuk D.V., Arsenyev A.I., Alexandrovna E., Tyuryaeva E.I., Antipov E., Girshovich M.M. (2023). Peripheral blood lymphocyte changes after stereotactic ablative body radiotherapy to lung or liver metastases in patients with oligometastatic cancers. Radiat. Oncol. J..

