# Supplementary material for: IFN-Type-I Response and Systemic Immunity in Rectal Adenocarcinoma Patients Treated with Conventional or Hypofractionated Neoadjuvant Radiotherapy
Source: Biomolecules. 2024 Apr 6;14(4):448. doi: 10.3390/biom14040448 (PMC11048635; doi:10.3390/biom14040448)
Supplement: Supplementary file 1 [file biomolecules-14-00448-s001.zip › biomolecules-2916574-supplementary.pdf]

Table S1

	scRT	lcRT
No. patients	22	40
Gender		
Male	14	31
Female	8	9
Age		
Range	52-85	28-87
Median	68	71
PS		
0	19	33
1	3	7
Histological type		
Adenocarcinoma NOS	22	40
Distance from anorectal ring (cm)		
Range/median	1-12/5	1-12/4
cT stage (MRI)		
T2*	1	0
T3	21	40
cN status (MRI)		
Positive	21	36
Negative	1	4
Tumor regression grade (TRG) AJCC/CAP**		
0	3	4
1	7	4
2	10	24
3	2	8

Figure S1

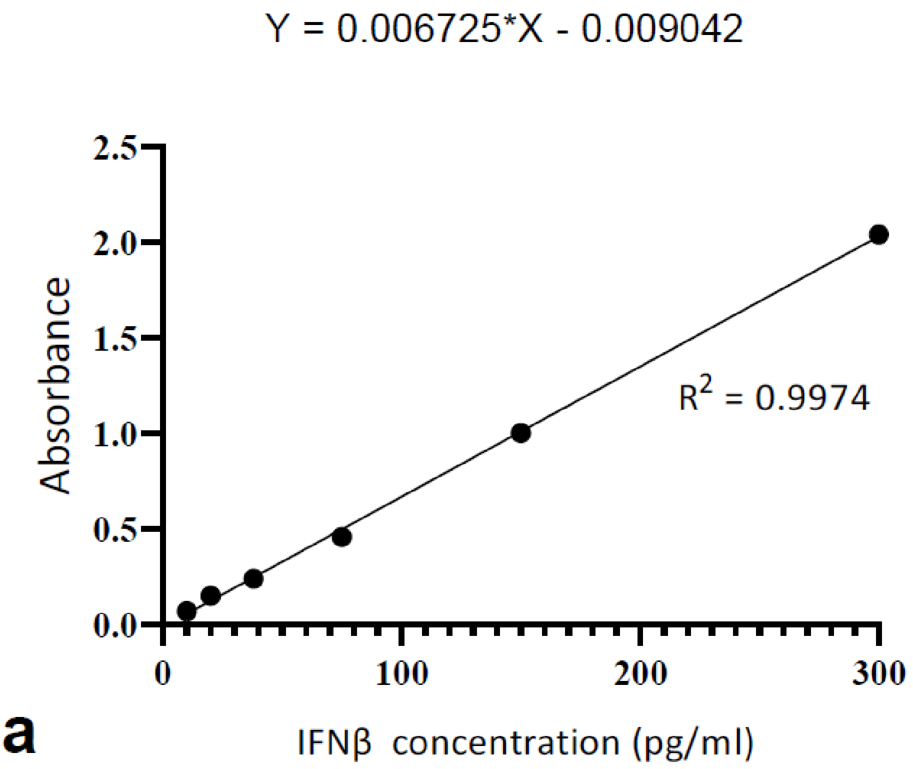
Y = 0.006725*X - 0.009042
Absorbance
2.5
2.0
1.5
1.0
0.5
0.0
0
100
200
300
R2 = 0.9974
IFNβ concentration (pg/ml)

a

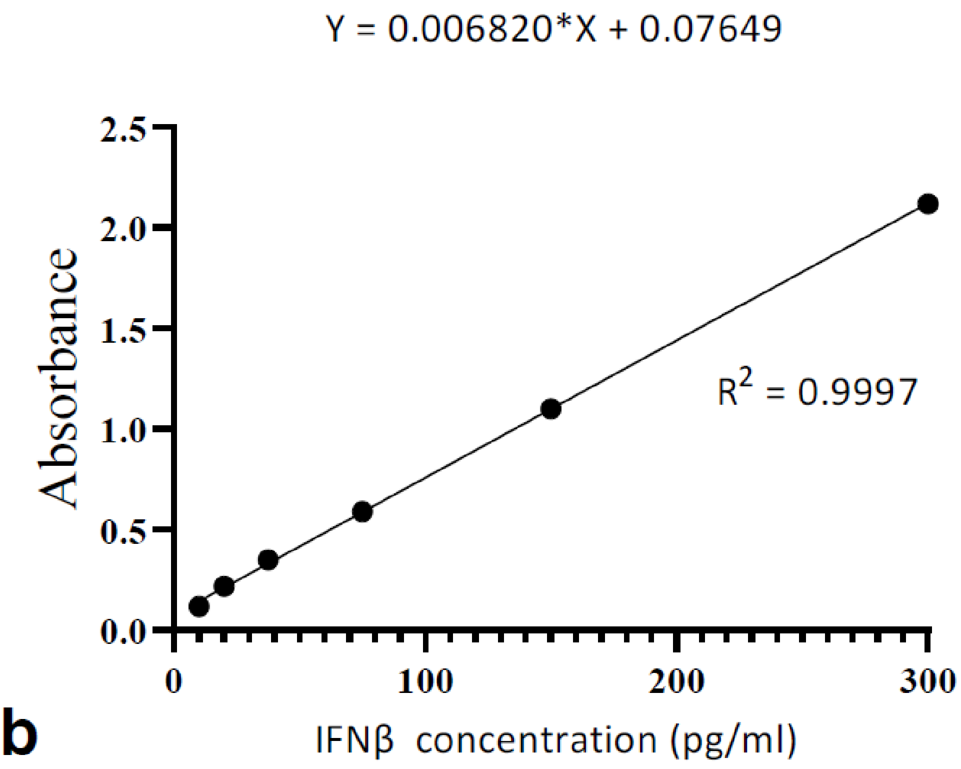
Y = 0.006820*X + 0.07649
Absorbance
2.5
2.0
1.5
1.0
0.5
0.0
0
100
200
300
R2 = 0.9997
IFNβ concentration (pg/ml)

b
